# Does Trait Affect Impact Activity Engagement?

**DOI:** 10.1093/geroni/igaa057.1259

**Published:** 2020-12-16

**Authors:** Rebekah Knight, Allura Lothary, Thomas Hess

**Affiliations:** North Carolina State University, Raleigh, North Carolina, United States

## Abstract

Research has shown the amount of effort we expend towards our goals depends on a sense of self-efficacy, perception of task difficulty, and likelihood of achieving our goal. All of these processes are susceptible to the influence of affect. For example, negative moods may impede goal achievement by increasing perceptions of difficulty (Silvestrini & Gendolla, 2019). Negative experiences (such as past failures) can encourage these negative moods and subsequently impact self-efficacy (Esposito, Gendolla, & Van der Linden, 2014). Findings from self-efficacy research (e.g. Esposito et al., 2014) suggest that older adults may be particularly susceptible to the impacts of negative affect on effort mobilization, especially when tasks already seem challenging, with little chance of success. Perception of task difficulty, then, impacts the amount of effort exerted in completing the task. The present study sought to examine the factors that impact perceptions of difficulty and subsequent effort expenditure, represented by systolic blood pressure responsivity (SBP-R). Younger (N = 41) and Older (N = 163) adults completed a difficult cognitive task as part of a larger, longitudinal study, as well as measures of trait affect before study sessions. Our findings indicate younger adults exert less effort overall than older adults; however, when negative trait affect is considered, we find that higher levels of negative affect in older adults reduced task engagement. These results provide support for an effect of negative affect on task appraisals and posited age-related differences in effort mobilization.

